# *“What would be left of me?”* Patient perspectives on the risks of obesity treatment: An innovative health initiative stratification of obesity phenotypes to optimise future obesity therapy (IMI2 SOPHIA) qualitative study

**DOI:** 10.1016/j.obpill.2024.100129

**Published:** 2024-08-23

**Authors:** Emma Farrell, Joseph Nadglowski, Eva Hollmann, Carel W. le Roux, Deirdre McGillicuddy

**Affiliations:** a University College Dublin, Dublin, Ireland; b Obesity Action Coalition, Tampa, FL, USA; c School of Medicine, University College Dublin, Dublin, Ireland; d School of Education, University College Dublin, Dublin, Ireland

**Keywords:** Obesity, Treatment, BMI, Weight management, Access, Bariatric, Diet, Patient voice

## Abstract

**Background:**

The uptake of obesity treatments remains disproportionally low in people living with the disease, even with the advent and availability of GLP-1 agonists in recent years. Efforts to understand this discrepancy have centred on literature syntheses and Healthcare Professionals’ (HCPs) perspectives on the barriers to obesity treatment. This study focuses on patient perspectives on the *risks* of obesity treatment.

**Method:**

This qualitative study consisted of online focus groups with 30 adults with obesity from Europe and North America. The focus group discussions were recorded, transcribed verbatim and analysed thematically.

**Results:**

Patients identified three risks associated with obesity treatment: (a) the risk that they can’t access treatment; (b) the risk that they would fail to meet treatment expectations – their own, their HCPs and societal expectations, and (c) the risk that the treatment would be ‘successful’ but that they would lose their sense of self, their coping mechanisms and identity along with weight.

**Conclusion:**

Understanding patient concerns about the risks of obesity treatment is essential to addressing obesity treatment inertia.

## Graphical abstract

**Figure:**
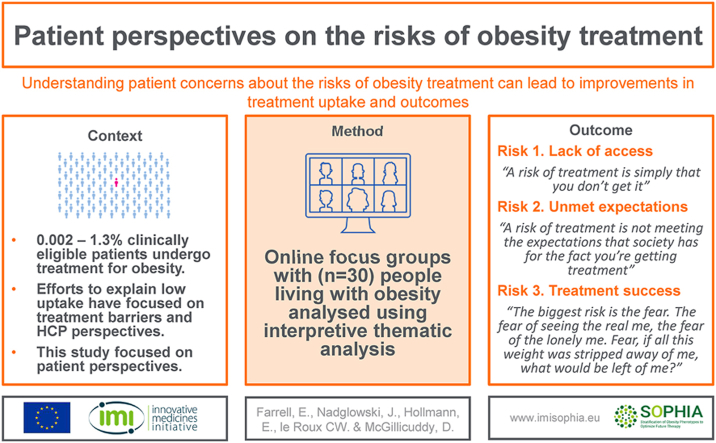

## 1 Introduction

Obesity is a disease of global concern and is associated with a range of negative health outcomes [1,2]. A range of evidence-based treatment options now exist for the management and treatment of obesity. These include lifestyle interventions (e.g. nutrition, physical activity), psychological therapies, pharmacologic and surgical interventions [3]. Despite evidence for the effectiveness of these treatments in the management of obesity, the uptake of obesity treatments, particularly non-lifestyle interventions, remains proportionately low. For example, a cohort study by Saxon et al. [4] found that just 1.3 % of 2.2 million patients in the USA who met the National Institutes of Health eligibility criteria for weight-loss medication received a prescription for one of these medications. A study of US Veterans Health Administration patients found that even fewer, just 0.2 %, of the 2 million eligible patients filled a prescription for weight-loss medication [5]. Bariatric surgery is considered an effective treatment of obesity, resulting in larger and longer-lasting weight loss than other treatment types [6]. However, it is estimated that only 1 % of the clinically eligible population undergoes surgical treatment for obesity in the US [7,8]. Data from the 2014 Health Survey for England suggests that this percentage is even lower in the UK with just 0.002 % of those eligible undergoing bariatric surgery [9]. The introduction of GLP-1 agonists to the obesity treatment market in recent years has resulted in a dramatic increase in uptake [10]. It is estimated that by 2030 9 % of the US population will be GLP-1 users [11]. However, given the prevalence of the disease, which the US Centres for Disease Control and Prevention (CDC) currently posits at 41.9 % [12], a gap remains between those eligible for, and the number of people accessing, obesity treatment.

Efforts to explain this gap typically take the form of review articles that synthesise the best available literature. For example, Forgione et al. [13] in a review of barriers to obesity treatment propose a four-stage plan to overcome these barriers in primary care based on a synthesis of literature. Busetto et al. [14], in a discussion of treatment inertia in obesity as compared to other non-communicable diseases, draw on literature relating to obesity stigma, prevention and treatment in suggesting that the simplistic narrative of obesity as a self-imposed condition with an easy way out (“eat less, move more”) results in limited or delayed treatment adoption by both patients and healthcare professionals. Moreover, Mauro et al. [15], identified (a) a lack of healthcare professional (HCP) and patient knowledge about obesity as a disease, (b) socioeconomic status, (c) time constraints, (d) saboteurs (e) comorbidities, (f) medication and, (g) alcohol and substance abuse as major barriers to obesity treatment and treatment effectiveness. It is worth noting that, of the 88 research papers cited in this review, just one involved interviewing patients to garner their perspectives as part of a mixed methods study on physical activity. In the years since the publication of Mauro et al.’s, review there has been greater recognition of the importance of patient voice in understanding and improving obesity treatment access and outcomes. Indeed, it has been systematically highlighted just how few studies are available to offer insight into patient perceptions and experiences of obesity and obesity treatment [16].

Understanding patient lived experience drives improvements in treatment design, quality and outcomes [17]. It offers clinicians and research scientists a rich understanding of the positive and negative aspects of the patient journey and enables them to address concerns that patients might have about obesity treatment. Insights that emerge from patient experience research are primarily rooted in qualitative data [18]. This study sought to qualitatively understand the risks of obesity treatment from the perspective of people living with obesity.

## 2 Methods

### 2.1 Study design

The data shared in this paper were generated through a series of focus groups with adults living with obesity. In contrast to individual or group interviews, focus groups encourage interaction between participants: asking questions, sharing experiences, and expanding similarities and differences in points of view. As such, focus groups are particularly effective in exploring people’s knowledge and experiences and offer an insight into, “not only what people think, but how they think and why they think that way” [19].

### 2.2 Recruitment and participants

Participants were recruited on the basis of experience of obesity treatment. We sought to include participants at the early stages of their treatment journey, as well those more seasoned in accessing support and care for obesity. To do this, we used convenience sampling to recruit participants at the early stages of their engagement with one Irish hospital-based weight management clinic (n = 7), as well as participants from leading European and US obesity advocacy organizations Obesity Action Coalition (OAC) and European Coalition of People living with Obesity (ECPO) (n = 23). This involved engaging a representative from each organisation who kindly extended an invitation to members to participate in this research. This invitation included contact details for the research team and interested participants themselves made contact to participate. The sample included 11 men and 19 women, of which 24 were European and 6 based in the US or Canada. The research team was interested to note that, while each country has its own approach to the provision of obesity treatment, the risks identified by participants, as presented below, were largely the same. Table 1 provides further detail on the sample as well as an overview of the treatments access by participants. Each participant has been assigned a pseudonym in order to strike a balance between protecting their anonymity and preserving their unique and individual character.

**Table 1:** Sample characteristics and overview of treatments undertaken by participants.

	Sex	Age range	Self-directed Behaviour Modification	HCP-Led Behaviour Modification	Meal Replacements	Community-Based/Non-Medical Programmes	Commercial Weight Loss Programmes	Supplements	Medications (prescribed)	Bariatric surgery
Amanda	F	40–49	X	X	X	X	X		X	X
Anna	F	40–49					X			X
Bob	M	60–69	X	X	X					X
Brianna	F	50–59	X	X	X	X	X		X	X
Caroline	F	50–59								X
Camila	F	70–79	X	X	X	X	X			
Damian	M	50–59						X	X	
Daniella	F	50–59	X	X	X	X	X	X		
Eddie	M	40–49	N/A	N/A	N/A	N/A	N/A	N/A	N/A	N/A
George	M	60–69	X	X	X	X			X	
Graham	M	60–69	X	X	X	X	X			X
Harper	F	40–49							X	
Isla	F	50–59	N/A	N/A	N/A	N/A	N/A	N/A	N/A	N/A
Kimberly	F	40–49	X	X	X	X	X	X		X
Layla	F	30–39	N/A	N/A	N/A	N/A	N/A	N/A	N/A	N/A
Lydia	F	40–49	X	X		X	X		X	
Melanie	F	30–39	X	X		X	X	X	X	X
Max	M	50–59	X	X		X	X	X	X	X
Maeve	F	60–69				X			X	X
Matteo	M	30–39	N/A	N/A	N/A	N/A	N/A	N/A	N/A	N/A
Olivia	F	30–39	X	X		X	X			X
Ronan	M	40–49	X	X			X		X	X
Samantha	F	20–29							X	X
Stella	F	50–59		X					X	
Selina	F	30–39							X	X
Susanna	F	50–59	X	X	X	X	X	X	X	
Sonia	F	40–49	X	X	X	X	X	X	X	X
Taylor	M	60–69		X						
Vihaan	M	30–39	X		X	X		X		
Walter	M	70–79	X	X	X					

Note N/A means that this participant chose not to complete the voluntary questionnaire. Participants who did not complete the questionnaire provided detail about their obesity treatment in the focus group interviews.

### 2.3 Data generation

Online focus groups were carried out with participants between April and July 2022. Each participant engaged in one focus group, with group sizes ranging in size from two to six persons depending on participant availability. Focus groups were scheduled at a time convenient for participants and their respective time zones. The purpose of the focus group was to understand patients' experience of obesity treatment and, in this instance, specifically their concerns about the risks associated with obesity treatment. The risks of obesity treatment was identified as a subject warranting further examination during earlier phenomenological data generation with a different group of people living with obesity [20]. Each group was offered the same question – what do you think are the greatest risks of obesity treatment? Occasionally participants would ask for clarification about what we meant by ‘risk’ but the interviewers were instructed to encourage an open interpretation of ‘risk’ so as not to limit the conversation in any way. This resulted in a broad conversation that encompassed, not just the very valid “side effects and stuff” (Samantha), but the more nuanced exploration of patient concerns that are reflected in the data below. In advance of each focus group, participants were sent an information sheet and consent form which they had time to read and discuss with researchers if they had any questions or concerns. Participants were also invited to complete a brief online questionnaire which asked them to indicate the types of treatment they have tried to date. These data are presented in Table 1.

### 2.4 Data analysis

All eight online focus group discussions were recorded and transcribed verbatim and initial analysis completed by one researcher using Braun and Clarke's [21] six-step framework for thematic analysis. This framework involved (a) becoming familiar with the data by reading and rereading the data in their entirety; (b) generating initial codes by jotting down descriptive labels and key words; (c) taking these initial codes and sorting them into potential themes; (d) reviewing and refining these prospective themes in terms of how accurately they reflected the meanings in the data; (e) further refining and defining these themes and what they revealed about participants perceptions of the risks associated with obesity treatment; and (f) formulating the findings as a ‘whole’ and presenting them as described below. The wider research team were presented with findings at step (d) and step (f) and the ensuing discussion facilitated a degree of inter-rater reliability and honed the thematic findings presented below.

## 3 Results

Patients perspectives of the risks associated with obesity treatment can be described under three headings: treatment access, treatment expectations and treatment success.

### 3.1 Treatment access

The most commonly cited risk associated with obesity treatment was, as Lydia highlights, that you simply don’t get it:

A risk of treatment is simply that you don’t get it – Lydia

Barriers to treatment identified by participants included lack of reimbursement in the public sector, lack of insurance cover, long waiting lists and narrowly defined eligibility criteria.

Lydia, for example, went on to explain how her private health insurance foreclosed any form of treatment for obesity – treatment she would be able to access had she another chronic disease such as diabetes.

“One of the realities of my own situation right now is, I have insurance through my employer that denies bariatric coverage in any sense. Any bariatric coverage of any flavor, that includes behavioral health, that includes nutritionist, that includes bariatric surgery, that includes pharmaceuticals, that includes anything. If I were diabetic, I could get access to some of those things – I don’t happen to be diabetic, so I am not eligible for my insurance to help support treatment that would help me treat my disease – and that is a reality for a lot of people in the United States” – Lydia

Kimberly also considers lack of access to treatment to be the greatest risk for people living with obesity.

“I think the roadblocks to treatment that are greater than the risks of treatment itself” – Kimberly

Being “shut down” by healthcare providers, a lack of private insurance cover and strict weight loss requirements were just some of the roadblocks Kimberly identified:

“I think that the issue is going to receive treatment and being shut down. Or, going to seek a therapy and it’s not covered by your insurance. Or, well we know that the only thing that had been proven to lower you set point is medication or surgery. So, why do people have to suffer through six months of a 1200- calorie diet plan that doesn’t work, just to get to the thing that does?” – Kimberly

Waiting lists for obesity treatment in the public sector were also identified as a major barrier.

“If you look for treatment, you’re on a list for five years before you have an appointment” – Maeve

And for those who do access treatment, the benefits of treatment, such as a reduction in BMI, itself makes them ineligible to continue accessing the treatment that has had such a positive impact on their quality of life.

“I live with obesity, I get access to care, but what happens once I’m on my best weight? I no longer qualify, to have access to the treatment” – Matteo.

### 3.2 Treatment expectations

One of the most frequently identified risks associated with obesity treatment is that of unmet expectations.

“A risk of treatment is not meeting the expectations that society has for the fact you’re getting treatment” - Selina

The main expectation participants’ spoke about was the expectation they would lose weight, and maintain weight loss, directly as a consequence of treatment. This expectation came from healthcare providers, family and friends, and wider society.

Selina described how, when she would attend her healthcare appointments:

“people would say, ‘Oh you’re Dr [name]’s patient, so you’re going to have surgery’ and I said ‘no, I've already had it’[…] it's just hard. I was supposed to lose this weight and keep it off - and it's an expectation” - Selina

Selina went on to describe the promises contained in commercials and advertisements for obesity treatments in the US and how they created the expectation that “your life’s just going to be so much better”:

“You see the commercials … all the before and afters, and your life’s just going to be so much better, you’re going to be so much happier, you’re going to meet someone and be able to have kids, and all this stuff is just … and then when that doesn't happen for people …” - Selina

Harper too described the reality that “nothing works for everybody, you know” and the demoralising effect of unmet treatment expectations:

“There's a risk that it won't meet either the doctor, or the patient's expectations. So, like you could - the patient could still feel that demoralized you know. If it didn't work or if didn't work, maybe, as well as it did for the next two people in the clinic, then it can be very demoralizing” - Harper

Maeve too warned about the expectation of dramatic and lasting results from obesity treatment:

“You think you’re fixed with the surgery – you’re not, you’re never fixed.”

This was a common theme throughout the focus groups. In separate groups Anna, Samantha and Sonia all warned against the expectation of “a fix” or “a cure”:

“That perception of others – so, others see that I’ve just had a cure, and people do feel that if you’ve had treatment for obesity, how could you gain weight again?” - Anna

“The other risk is people think it’s a fix. It’s not; it is a tool on a lifetime journey.” - Samantha

“I think when people hear the word treatment, they seem to think this is the fix. So, I think there needs to be more understanding that it's the treatment for now, but it doesn't mean it's ongoing.” - Sonia

Other expectations identified by participants included the expectation that “you should be able to do it yourself” (Layla) and the idea that “obesity is still seen as a lifestyle choice” (Max) and not a chronic disease deserving of treatment and support.

“I had the Roux-en-Y [surgery] so they were rerouting everything, they were cutting away a part of my small intestine, and they were rerouting my stomach - so incredibly dangerous. And yet, I took the ‘easy way out’” - Max

In addition to the expectations of others, participants described their own disappointment and frustration when treatment “doesn’t produce the results” (Vihaan) that they had hoped for.

“But then the danger, the risk of that, of course, is that when it [treatment] doesn't produce the same results for everybody, and then folks are starting to think like, ‘oh, well it worked for that other person, why didn't it work for me? Am I a failure? Did I fail this?’” - Vihaan

“But I’m telling you, it's frustrating to be on a medication, where you see everyone else post about the successes they're having and how much weight they're losing, and it took me almost a year to lose 20 pounds on it” - Selina

### 3.3 Treatment success

One of the, perhaps surprising, risks of treatment identified by participants was that the treatment might be successful.

Daniella says “I think the biggest risk is actually the fear”:

“The fear of actually seeing the real me, the fear of the lonely me […]. Fear, if all this weight was stripped away of me, what would be left of me?” – Daniella

She went on to describe the loss of comfort, the experience of life “stripped bare”:

“I’d have no comfort anymore. What am I going to reach to for comfort? It’s this fear, when everything is stripped bare and laid bare, what am I going to really see? All my comforts are taken away from me” – Daniella

Selina too spoke about how “difficult” it was when, as a result of obesity treatment, “food doesn’t comfort you the same way”.

“I think a lot of us, when food doesn't comfort you the same way, and you have to actually look and figure out coping skills, it can be difficult” – Selina

Participants described one of the greatest risks of treatment being that they would be treated differently, that they would suddenly become “far more acceptable” because of treatment and, as Brianna put it, “that’s not OK”. Brianna and Ben discussed this in one focus group and offer an insight into how weight loss, seemingly an intended consequence of treatment, can have unintended, and unappreciated, consequences.

Brianna

“The fact that you get treated so differently by even the same people, it kind of makes you question … obviously the weight being gone kind of makes you look different, but you’re not a different person than you were. You’re not more intelligent – you are who you were. If you were kind, you’re still kind. If you weren’t kind, you’re still not kind. [Treatment] doesn’t alter your personality and yet for some reason you’re far more acceptable and that’s not OK”

Bob

“It does alter the way you’re treated; I mean you go to work, you will be seen like, people with obesity will be seen as being lazy and stupid”.

Brianna

“Exactly. And then suddenly you’re not”.

Olivia too described a change in relationships, saying she “lost a few friends” as a result of her post-surgery weight loss.

“The risks [associated with obesity treatment]? Your friendships change, everything in life changes. Like, people going, no friendships don’t change – they do – Olivia

While access, expectations and success were all identified by people living with obesity as the greatest risks of obesity treatment, the majority retuned to the point that “it is definitely worth it” (Vihaan).

It's important to look at the other side of the risk, and that's the risk of not taking action - Vihaan

## 4 Discussion

Patients who participated in this study identified three risks associated with obesity treatment: treatment access, treatment expectations and treatment success.

As Lydia summed up effectively – the preordinate risk of obesity treatment is “simply that you don’t get it”. Barriers to treatment identified by the 30 participants in this study include certain treatments just not being available – particularly to those where public provision is poor or those without health insurance or the financial means to pay for treatment. While the form of this particular barrier varied slightly from country to country, the common theme of affordability ran through the accounts – those who could afford the treatment, or whose health insurance covered the cost, were much more likely to access treatment for their obesity. Since as far back as 2014, evidence has clearly suggested if governments invest in funding obesity treatments, the healthcare costs associated with obesity could be reduced by as much as 55 % [22]. Participants described long waiting times, being “shut down”, as Kimberly put it, by healthcare providers with limited understanding of obesity as a disease, or treatment protocols that require patients “to suffer through six months of a 1200 calorie diet plan that doesn’t work to get the thing that does”. The legacy of the simplistic “eat less, move more” narrative of obesity treatment certainly appears to be a factor delaying or restricting access to treatment for those who participated in this study. For those who do access treatment, there is always the risk, as Matteo highlights, of being ineligible for the treatment when they reach their “best weight” thereby placing them at risk of losing the initial health gains. It should also be noted that, as evidenced in Table 1., participants in this study have all successfully accessed obesity treatment and many have accessed multiple forms of treatment at different points in time. The voices and experiences of those who failed to overcome the barriers to treatment access are not represented here and the findings of this study suggest that these warrant further attention.

The expectations surrounding obesity treatment, one’s own expectations and those of others, also emerged as a point of vulnerability for those seeking and accessing treatment for obesity. The main expectation was that obesity treatment would result in weight loss. Even more than that, as Selina says, “I was supposed to lose this weight and keep it off”. The reality that “nothing works for everybody”, as Harper put it, came through in the accounts of those who participated in this study and the difficulty they had in managing their own expectation, and those of others, if weight loss was not an immediate and long-lasting consequence of treatment. Research shows that 15 % of patients do not respond to weight loss medications [23] and many of those who do will regain a substantial amount of weight if they stop taking GLP-1 agonists [24]. Further, amongst bariatric surgery patients, considered the “gold standard” of obesity treatments, a weight loss failure rate of 11.2 % has been recorded [25]. Participants described the expectation that obesity treatment is “a cure” (Anna), that “you’re fixed” (Maeve, Samantha, Sonia) as soon as you undergo treatment, to be a difficult one to manage – for themselves, their HCPs, families and society. Vihann and Selina spoke about the sense of failure and the frustration when a treatment “doesn’t produce the same results for everybody”. It can be “very demoralising” for patients as they come to terms with the reality that “it didn’t work” (Harper).

For those who do experience the results they desired, there is the reality, as Sonia put it, that “it’s the treatment for now”. As Samantha says “people think it’s a fix. It’s not; it is a tool on a lifetime journey” and adjudging to the lifetime nature of that journey was experienced by participants as an unexpected challenge of obesity treatment. These expectations, and the risk they pose to treatment uptake and adherence, will only increase in light of the public narrative of success that surrounds the release of obesity medications.

The third risk associated with obesity treatment was that it might be successful. Success, in obesity treatment terms, is often, rightly or wrongly, defined in terms of weight loss or a reduction in Body Mass Index (BMI). While there has been growing emphasis on defining obesity treatment success in terms of health gain rather than weight loss [26,27], weight loss, for participants, remailed the primary focus of obesity treatment. Weight loss, as participants in this study voiced so clearly, is associated with more than just a reduction in body mass. There is an emotional component to weight and losing weight is associated, as Daniella described, with fear: “the fear of actually seeing the real me, the fear of the lonely me […]. Fear, if all this weight was stripped away of me, what would be left of me?”. The exposure described by Daniella, the fear of being “stripped bare” and the loss of comfort when all her comforts are “taken away” highlights just how deep seated and profound the loss associated with weight loss can be. Daniella was not alone in voicing this fear. Selina too spoke of the loss of comfort, the exposure and sense that she had “to actually look” at herself and her life following weight loss.

While for many living with obesity weight loss has a positive influence on mental health, studies have found increased incidence of depression, self-harm and suicide in patients undergoing bariatric surgery. One systematic review suggests that suicide risk for patients undergoing bariatric surgery is four times higher than the general population norm. The temporal proximity of these suicide to bariatric surgery indicates a causal relationship. A number of theories have been presented to account for this increase in psychological distress. These include the unmet expectation that life will dramatically change after surgery [28]; increased stress and anxiety [29]; increased substance abuse [30] and the impact of treatment on emotional eating [31] - having this coping mechanism “taken away” as Danielle described. It is notable that participants in separate focus groups independently identified the psychological and emotional effects of obesity treatment as a “risk”. While epidemiological data notes an increased incidence in depression, self-harm and suicide post bariatric surgery, these “bottom up” data suggests that the psychological impact of treatment is a pressing concern for people living with obesity – one that warrants further, qualitative, attention. These data reinforce the importance of psychological and emotional support for patients who undergo obesity treatment. They also emphasise the importance of listening to patients’ concerns and monitoring the reality that, while obesity treatment “is definitely worth it” as Vihann says, it is not without risk.

### 4.1 Limitations

A limitation of this study is its sample size and representativeness. Participants were selected on the basis that “they might have something to say about an experience they share with others” [32]. This non-probabilistic nature of this convenience sample, combined with a small sample size (n = 30), means that the study’s findings are not generalisable. However, as the aim of this study was to generate rich, patient-led, explanatory data, generalisability was of minimal concern. Indeed “transferability” has been deemed a more appropriate measure of *probabilitas* or authority in qualitative research.

## 5 Conclusion

This study examined the risks of obesity treatment from the perspective of people living with obesity. Its aim was to qualitatively understand patient concerns with a view to better understanding the discrepancy between the number of people who access obesity treatment relative to the number who are eligible for it. While a lack of access to treatment was a major concern for people living with obesity, the risk of unmet expectations and the psychological and emotional consequences of treatment “success” also emerged as considerable risks for patients. This study highlights the importance of a multi-disciplinary approach to obesity treatment, one that includes psychological and emotional support, and the need to listen to the concerns of people living with obesity in developing effective responses to the treatment of this disease.

• Lack of treatment access due to insurance limitations, long waiting lists, narrowly defined eligibility criteria and a lack of long-term treatment options, is the greatest treatment risk for people living with obesity.
• Treatment success presented a risk for some people living with obesity. The change in identity, sense of self, physical weight and the loss of food-related “comfort” and “coping mechanisms” left many patients feeling “stripped bare”. This emergent theme may offer insight into the known, but poorly understood, increased incidence of depression, stress, anxiety and suicide following obesity treatment.
• This study highlights the importance of understanding patient concerns about the risks of obesity treatment as part of an effective treatment approach.

## Author contributions

All authors were involved in the conception and design of the study. EH recruited participants. EF and EH conducted the focus groups. EF performed the analysis and is guarantor. EF drafted the manuscript with review and approval for submission from JN, CLR and DMcG. The corresponding author attests that all listed authors meet authorship criteria and that no others meeting the criteria have been omitted.

## Ethical statement

The study was approved by the UCD Human Research Ethics Board Committee and conducted in accordance with the principles of the Helsinki Declaration (HS-20-

12-McGillicuddy). Participants were informed they could withdraw at any stage without penalty. Written informed consent was obtained from all participants.

## Funding statement

This study is part of the SOPHIA project which received funding from the Innovative Medicines Initiative 2 Joint Undertaking under grant agreement No 875534. This Joint Undertaking support is from the European Union’s Horizon 2020 research and innovation programme and EFPIA and T1D Exchange, JDRF, and Obesity Action Coalition.

## Declaration of artificial intelligence (AI) and AI-assisted technologies utilized in the writing process

Artificial intelligence (AI) and AI-assisted technologies were not used in the writing or preparation of this manuscript.

## Declaration of competing interest

Authors declare support from the Innovative Medicines Initiative 2 for this research. The authors declare no financial relationships with any organizations that might have an interest in the submitted work in the previous 3 years, and no other relationships or activities that could appear to have influenced the submitted work. Le Roux reports grants from the Irish Research Council, Science Foundation Ireland, Anabio and the Health Research Board. He serves on the advisory boards of NovoNordisk, Herbalife, GI Dynamics, Eli Lilly, Johnson and Johnson, Sanofi Aventis, Astra Zeneca, Janssen, Bristol-Myers Squibb, Glia, and Boehringer-Ingelheim. He is a member of the Irish Society of Clinical Nutrition and Metabolism outside the area of work commented on here, He is also the Chief Medical Officer and Director of the Medical Device Division, and is a previous investor in Keyron, which develops endoscopically implantable medical devices intended to mimic the surgical procedures of sleeve gastrectomy and gastric bypass, He divested stock holdings in Keyron in September 2021.
